# Fractures of the Craniofacial Skeleton in the Elderly: Retrospective Studies

**DOI:** 10.3390/ijerph182111219

**Published:** 2021-10-26

**Authors:** Piotr Michalak, Grażyna Wyszyńska-Pawelec, Mariusz Szuta, Justyna Hajto-Bryk, Jan Zapała, Joanna Katarzyna Zarzecka

**Affiliations:** 1 Department of Conservative Dentistry with Endodontics, Jagiellonian University Medical College, 31-008 Krakow, Poland; justyna.hajto@uj.edu.pl (J.H.-B.); j.zarzecka@uj.edu.pl (J.K.Z.); 2 Department of Cranio-Maxillofacial Surgery, Jagiellonian University Medical College, 31-008 Krakow, Poland; grazynawyszynska@wp.pl (G.W.-P.); jan.zapala@uj.edu.pl (J.Z.); 3 Department of Oral Surgery, Jagiellonian University Medical College, 31-008 Kraków, Poland; mariusz.szuta@uj.edu.pl

**Keywords:** aged, aging, older people, gerodontology, oral health, fractures, maxillofacial injuries, retrospective studies

## Abstract

The aim of the retrospective analysis of the medical documentation of 101 patients was to assess the incidence, etiology, and type of craniofacial fractures in the elderly population of southern Poland, who required specialist treatment at the Department of Cranio-Maxillo-Facial Surgery Jagiellonian University, Krakow, Poland, in the period 2010–2019. Patients were divided into 3 age groups: 65–74, 75–84, and 85 and older. The following was noted: age, sex, place of residence, education, cause and location of fracture, treatment, injuries and comorbidities, complications, alcohol and other drugs at the time of injury, and the period of hospitalization. The dominant group were patients aged 65–74 (72.28%), mainly males (56.44%). The main cause was fall (47.52%). The fractures involved mainly the mandible and the zygomaticomaxillary complex. Over half of patients (50.50%) lived in the countryside or small towns. Work tool-related accidents prevailed among geriatric patients living in small towns and rural areas. Craniofacial fractures were additionally accompanied by common complications regarding the organ of vision. Further studies analyzing factors leading to increased risk of craniofacial injuries in the elderly of the rural population will enable proper support programs, prophylaxis, and principles concerning agricultural activities.

## 1. Introduction

Craniofacial fractures are a significant health issue. With increase in life expectancy, a rise in the frequency of craniofacial injuries occurs [1]. In geriatric patients, progressive aging of the body, frailty syndrome, senile changes in the nervous system lead to frequent falls and serious injuries with higher risk of complications, including death, in comparison with the younger patients [2,3].

According to the literature, midface fractures are the most common fractures of the facial skeleton affecting elderly population [3,4,5]. Comminuted fractures accompanied by edema, hemorrhage, displacement, and mobility of thin bone fragments lead to masticatory dysfunction, dysphagia, and intense pain. Due to multiple comorbidities, anticoagulant therapy, and age-related vascular fragility, post-traumatic hematomas, sometimes life-threatening, e.g., impairing respiration, might develop quickly [6]. 

In geriatric patients, significant deterioration in the quality of life occurs immediately following the injury. The likelihood of developing post-traumatic emotional disorders after craniofacial fractures, including depression, amounts from 20% to 41%. Ageism-related behavior and loss of independence may impact the convalescence and prevent full recovery of geriatric patients suffering from the fractures of the facial skeleton [7,8].

The aim of this study was to conduct a comprehensive review of craniofacial fractures in the elderly population of southern Poland, who required specialist treatment at the Department of Cranio-Maxillo-Facial Surgery of the Jagiellonian University, Krakow, Poland in the period 2010–2019.

## 2. Materials and Methods

A retrospective analysis of the medical documentation of 101 patients with craniofacial fractures reporting to the Department of Cranio-Maxillo-Facial Surgery of the Jagiellonian University, Krakow, Poland, in the years 2010–2019, was performed. The study included people aged 65 and older at the time of injury, who required hospitalization due to fractures in the craniofacial region. Patients were divided into 3 age groups: 65–74, 75–84, and 85 and older. Owing to the decreasing number of patients related to their age, the group of 75–84-year-olds and 1 older patient were joined. The following was noted: age, sex, place of residence, education, cause of the fracture, location of the fracture, method of treatment, injuries and comorbidities, complications, alcohol and other drugs at the time of injury, and the period of hospitalization.

Comparison of the value of quality variables in the groups was performed using the chi-squared test (with Yates correction for tables 2 × 2), or Fisher’s exact test. Mann–Whitney U test was utilized to compare the value of quality variables in two groups. Significance level for the analysis amounted to *p* ≤ 0.05. The study was conducted with the consent of the Bioethics Committee of the Jagiellonian University; KBET UJ 1072.6120.101.2020.

## 3. Results

The dominant group were patients aged 65–74 (72.28%), mainly males 57 (56.44%). Over half of the patients (51 or 50.50%) lived in rural areas or small towns, with up to 20 thousand inhabitants, and received education at a secondary level (52.48%). All patients were retirees. Detailed characteristics of the patients are presented in Table 1.

The most common comorbidities observed included: cardiovascular diseases (83 or 82.18%) and the visual system diseases (26 or 25.74%). More than 1 comorbidity was declared by 71 of the patients (70.3%) (Table 2). The average number of medications taken amounted to 4.11 and ranged from 0 to 9.

The main cause of the injury was fall, constituting in total 48 (47.52%) of the cases, including a fall with loss of consciousness in 15 (14.85%), followed by work-related injuries in 12 patients (11.90%). There were 78.22% of the patients who denied alcohol consumption before injury. Detailed causes of the craniofacial fractures are shown in Table 3.

The fractures involved most often the mandible (63 or 62.38% of the cases) and the zygomaticomaxillary complex (ZMC) (39 or 38.61%). Fractures of the mandible mainly involved displaced fracture of the mandibular body Multiple fractures of the craniofacial skeleton constituted 55 of the cases (54.46%). The most frequent was simultaneous nasal and ZMC fracture, as well as mandible and maxilla or ZMC fracture. Craniofacial fractures in the analyzed material are presented in Table 4.

The most commonly observed injuries coexisting with the craniofacial fractures were soft tissues injuries and injuries of the visual system, 87.13% and 24.75%, respectively. Central nervous system injuries were presented only in 3 of the patients (2.97%) (Table 5).

Surgical treatment involving mini/micro plates osteosynthesis was conducted in general anesthesia in 80 patients (79.21%), including osteosynthesis of the mandible, ZMC, and maxilla, as well as revision of the orbital floor with bone grafting. Less invasive procedures, such as splinting or intermaxillary fixation, in cases of mandibular or maxillary fractures, were performed in local anesthesia in 23 (22.77%) of the cases. There were 48 patients (47.52%) who required hospitalization exceeding 7 days. The main long-term complications involved visual disturbances observed in 8 patients (7.92%). The majority of the patients (79 or 78.22%) were discharged in a local and general good condition, without complications (Table 6). 

The relationship between age, sex of patients, and location of the fractures was analyzed. Fractures of the mandible including displaced shaft fractures were significantly more frequent in the group of patients aged over 75 years old (*p* = 0.006). Table 7 presents detailed data.

Differences between the group of females and males reached a significant level only in the case of maxillary fractures, which were more common in males (*p* = 0.007) (Table 8). 

Cause of injury was analyzed in correlation with location of fracture, age, and place of residence of the patients. Significant correlation between the location of fracture and the cause of the injury was observed. Nasal (50%) and maxillary (58.33%) fractures were most often caused by a work tool. ZMC fractures significantly correlated with assault and traffic accidents (*p* < 0.001), whereas mandibular fractures as a pathological or iatrogenic fracture (Table 9). 

Falls were more common in patients over 75 years old than in the group of 65–74 years old, 53.57% and 45.21% respectively. However, we have not found any statistically significant correlation between the cause of fracture and age of patients (Table 10).

Work tool-related accidents took place only in small towns and rural areas and constituted 12 of the cases (23.53%). Falls occurred more frequently in medium-sized towns and cities, 64.28% and 63.88% respectively. Falls with loss of consciousness occurred mainly (35.71%) in medium-sized towns, which was statistically significant, as it is shown in Table 11. 

## 4. Discussion

The study conducted covered the analysis of 101 medical histories of patients who were at least 65 years old who required hospitalization due to craniofacial injuries. The discussion compares our own results and the results of other authors involved in the same issue among the elderly, despite the differences in defining the group in terms of the age. The age when people are classified as the elderly according to WHO is 60 in developing countries and 65 in developed countries, while the UN sticks to 60 years old [4,9]. 

The predominant age group in our study included people aged 65–74, (72.28%). Simultaneously, it was the youngest age group, although all patients were retirees. The life expectancy in Poland is relatively short, less than 84 years in women, less than 77 years in men which is consistent with demographics in our study group [10,11]. In Poland, the population of 85 and older is small. In the analyzed cohort, there was only one patient older than 85 years. The obtained results are contradictory to the data on geriatric patients with facial fractures from the USA, published by Marchini, where the largest group comprised patients aged 75–84 [12]. To support the analyses, it should be observed that the younger the age group included in the study, the more frequent injuries were observed in the group [13]. It most probably results from the decreasing physical activity, professional activity, lower mobility, and increase in the mortality rate. Increase in the frequency of craniofacial injuries is also observed in the elderly due to the prolonged life span and social campaigns aimed at their mobilization [1,12,13,14,15,16]. 

Analysis of gender revealed that the male group was bigger than the female one, which corresponds to other authors’ observations [3,4,13,14,17,18]. However, publications indicate a systematic increase in the number of women suffering from craniofacial fractures in the percentage share, especially in the older groups. It results from a longer life expectancy among women and an additional risk of fractures due to osteoporosis [3,19,20]. 

In our study, comparably to the studies conducted in other medical centers, the risk of craniofacial fractures was higher among residents of small towns and rural areas [15].

Attention was drawn to an increased risk of craniofacial injuries in the population of people with lower education level in the Brazil, where the frequency of craniofacial injuries caused by physical abuse was analyzed [21]. However, in our study group, patients with secondary and higher education predominated.

In our cohort, falls were the most common cause of injury. This is consistent with other publications, indicating the risk of facial injuries resulting from a fall in the geriatric population [2,3,4,5,12,14,18]. Falls are most often observed at the place of residence, mainly in bathrooms or toilets, from a height not exceeding 1 meter. The age of patients and height of the fall are inversely correlated [1,2,19,22]. Even low-energy falls are a more common cause of death in the elderly compared to younger people [8]. Cognitive dysfunctions, frailty, and unsteady gait may be related to falls in the elderly [17,23,24]. In our material, falls were observed more often in medium-sized towns and cities, whereas falls with loss of consciousness occurred mainly in medium-sized towns. That might be connected with social status of the elderly, who often live alone in urban areas and do not cope with everyday household duties [25].

Conducted analysis revealed no correlation between the cause of injury and age. Other authors indicate that the frequency of falls increases with age regardless of the sex, although there is a systematic increase in the number of women in the percentage share [19].

The next most common cause of facial fractures in our study was work tool-related injury, while other authors indicate traffic accidents [3,4,12,14]. The reasons may be found in low material status of the elderly in Poland and the resulting need for professional activity, especially in rural areas, despite the age worsening health condition. In our study group, over half of patients (50.50%) lived in villages or small towns. We observed a correlation between the cause of craniofacial fracture and the place of residence. Accidents with a work tool took place only in the rural areas and small towns. Studies by other authors, limited to the analyses of agricultural accidents, observed zoonotic injuries and, subsequently, work tool impact to be the most common cause [26].

The most common type of fracture in the analyzed cohort was fracture of the mandible and ZMC. Increased frequency of midface fractures was confirmed by other authors [3,4,5]. A minor share of nasal fractures (21.78%) in our material is characteristic and differentiating when it comes to other studies [2,3,4,13,17]. Patients with isolated nasal fractures, who were treated in an outpatient mode at the ER (Emergency Room) and, in the case of surgical intervention, were admitted to the Otolaryngology Department, were excluded from our study. According to the other authors, fractures within the craniofacial skeleton are most often multiple injuries, and half of the isolated injuries are limited to the nose [4].

In the current research, location of the fracture correlated with age. The risk of mandibular fracture, especially displaced, rose with age. It has been confirmed by other authors that the majority of the elderly people suffering from mandibular fractures are edentulous. Anodontia and bone atrophy result in the fact that mechanical forces act directly on the weakened bone, which may lead to pathological fractures [3,18].

In our material, correlation between the location of the fracture and the patient’s sex was found. Maxillary fractures were more frequent in men. Plawecki analyzed that aspect and demonstrated that nasal fractures are more frequent in women than men [13]. However, these results cannot be compared.

The current analysis revealed a correlation of the location and the cause of fracture. Nasal and maxillary fractures were mostly caused by a work tool, ZMC fractures were caused by assault and traffic accidents, and mandibular fractures were pathological or iatrogenic. Brazilian studies showed that the most frequent fractures concerned nasal bones (54%), mainly as a result of fall in the bathroom [2]. Analysis of the craniofacial fractures as a result of blunt injury demonstrated that more frequent fractures of the orbital floor, maxilla, and mandibular condyle in geriatric patients depend on the age rather than the mechanism of the injury itself [3].

The majority of the patients required surgical intervention (79.21%) in general anesthesia. The result corresponds to the studies by Patrick from Switzerland and Rui Li from China [5,14]. Low rate of conservative management compared to studies from other medical centers results from the exclusion of the isolated nasal fractures and soft tissue injuries, which are most often caused by low-intensity stimuli [4].

Patients in the current study demonstrated such complications as visual disturbances, sensory disturbances, and malocclusion. It should be noted that the majority of the patients were discharged home after hospitalization shorter than 7 days, without complications (78.22%). According to the literature, 44.4% of the elderly patients suffer from injuries coexisting with the craniofacial fractures, while, in the young patients, 25% of the cases [8]. Most frequently craniofacial injuries are accompanied by rib fractures and concussion, which are more common in the elderly. The frequency of spinal fractures coexisting with the craniofacial injuries depends on the mechanism of the injury. Failure to diagnose the injury, the so-called “undertriage”, is the cause of the complications and mortality among the elderly. Such serious injuries coexisting with facial fractures might be easily missed in geriatric patients due to difficult communication, e.g., problems with hearing and lack of witnesses of the accidents when elderly live alone [27].

In our cohort, none of the patients died. This study included patients with isolated craniofacial injury. Studies by other authors indicate that geriatric patients with general injuries require longer hospitalization in the Intensive Care Units and die more often due to the concomitant injuries than younger ones [3,4,8,12].

It should be stressed that the results presented in our study concern one center. Therefore, they reflect the specific nature of the area where our medical center is located.

## 5. Conclusions

In the elderly living in the southern Poland, falls were the most frequent cause of facial bones fractures. Mandibular and zygomaticomaxillary fractures were the most common. Work- tool related accidents prevailed among geriatric patients living in small towns and rural areas. Further studies analyzing factors leading to increased risk of craniofacial injuries in the elderly of the rural population will enable proper support programs, prophylaxis, and principles concerning agricultural activities

## Figures and Tables

**Table 1:** Characteristics of the study group.

Age	*n*	%
65–74	73	72.28
75–84	27	26.73
85 and older	1	0.99
**Sex**	***n***	**%**
Female	44	43.56
Male	57	56.44
**Place of residence**	***n***	**%**
Village, small town	51	50.50
Medium-sized town	14	13.86
City	36	35.64
**Education**	***n***	**%**
Elementary	17	16.83
Secondary	53	52.48
Higher	20	19.80
No data	11	10.89
In total	101	100

**Table 2:** Comorbidities in patients.

Comorbidities	*n*	% *
Cardiovascular diseases	83	82.18
Visual system diseases	26	25.74
Osteoarthritis	19	18.81
Diabetes mellitus	14	13.86
Neurological disorders	10	9.90
Pulmonary diseases	7	6.93
Cancer	6	5.94
Hepatic insufficiency	3	2.97
Chronic renal insufficiency	1	0.99
Hematopoietic system diseases	1	0.99
No comorbidities	9	8.91
Other	43	42.57
No data	2	1.98

* Percentage does not sum up as 100 since one may have multiple diseases.

**Table 3:** Cause of the fracture.

Cause of the Fracture	*n*	%
Fall	48	47.52
Work-related injury	12	11.90
Assault	11	10.89
Traffic accident	11	10.89
Pathological/iatrogenic fracture	11	10.89
Recreation	3	2.97
Environmental cause	2	1.98
Zoonotic injury	1	0.99
Gunshot injury	1	0.99
No data	1	0.99
In total	101	100

**Table 4:** Location of the fracture.

Location of the Fracture	*n*	% *
Mandible	63	62.38
Zygomaticomaxillary complex	39	38.61
Maxilla	34	33.66
Orbit	32	31.68
Nose	22	21.78
Multiple fractures	55	54.46
Other craniofacial bones	6	5.94

* Percentage does not sum up as 100 since one may have fracture in multiple locations.

**Table 5:** Injuries coexisting with craniofacial fractures.

Coexisting Injuries	*n*	% *
Soft tissue injuries	88	87.13
Visual system injuries	25	24.75
Cranial nerves injuries	13	12.87
Central nervous system injuries	3	2.97
None	13	12.87

* Percentages do not sum up as 100 as multiple injuries may be present in one patient.

**Table 6:** Method of treatment, length of hospitalization, and long-term complications.

Method of Treatment	*n*	% *
Osteosynthesis (micro/mini plates, general anesthesia)	80	79.21
Orthopedic (splints, intermaxillary fixation, local anesthesia)	23	22.77
Conservative	5	4.95
**Hospitalization**	***n***	**%**
Up to 7 days	53	52.48
Over 7 days	48	47.52
**Complications**	***n***	**%**
Visual disturbances	8	7.92
Sensory disturbances	7	6.93
Malocclusion	5	4.95
Infection	2	1.98
None	79	78.22%

* Percentage does not sum up as 100 since one may have multiple treatments.

**Table 7:** Location of the fracture and the patients’ age.

Location of the Fracture	Age		*p*
	65–74 (*N* = 73)	75 Years and Older (*N* = 28)	
Mandible	28 (38.36%)	20 (71.43%)	0.006 *
Zygomaticomaxillary complex	29 (39.73%)	10 (35.71%)	0.887
Maxilla	28 (38.36%)	6 (21.43%)	0.169
Orbit	26 (35.62%)	6 (21.43%)	0.257
Nose	19 (26.03%)	3 (10.71%)	0.162
Other craniofacial bones	5 (6.85%)	0 (0.00%)	0.318
Other	0 (0.00%)	1 (3.57%)	0.277

* Statistically significant; Chi-squared test or Fisher’s exact test.

**Table 8:** Location of the fracture and the patients’ sex.

Location of the Fracture	Sex		*p*
	Women (*N* = 44)	Men (*N* = 57)	
Mandible	23 (52.27%)	25 (43.86%)	0.523
Zygomaticomaxillary complex	16 (36.36%)	23 (40.35%)	0.84
Maxilla	8 (18.18%)	26 (45.61%)	0.007 *
Orbit	16 (36.36%)	16 (28.07%)	0.501
Nose	8 (18.18%)	14 (24.56%)	0.598
Other craniofacial bones	2 (4.55%)	3 (5.26%)	1
Other	0 (0.00%)	1 (1.75%)	1

* Statistically significant; Chi-squared test or Fisher’s exact test.

**Table 9:** Location and cause of fracture.

Location of the Fracture	Cause of the Fracture						*p*
	Fall (*N* = 48)	Assault (*N* = 11)	Traffic Accident (*N* = 11)	Work-Related Injury (*N* = 12)	Pathological/Iatrogenic Fracture (*N* = 11)	Other (*N* = 7)	
Mandible	20 (41.67%)	7 (63.64%)	4 (36.36%)	3 (25.00%)	10 (90.91%)	3 (42.86%)	0.018 *
Zygomaticomaxillary complex	14 (29.17%)	8 (72.73%)	8 (72.73%)	6 (50.00%)	0 (0.00%)	3 (42.86%)	<0.001 *
Maxilla	11 (22.92%)	5 (45.45%)	6 (54.55%)	7 (58.33%)	1 (9.09%)	3 (42.86%)	0.029 *
Orbit	17 (35.42%)	3 (27.27%)	4 (36.36%)	3 (25.00%)	1 (9.09%)	3 (42.86%)	0.58
Nose	8 (16.67%)	2 (18.18%)	4 (36.36%)	6 (50.00%)	0 (0.00%)	2 (28.57%)	0.039 *
Other craniofacial bones	2 (4.17%)	1 (9.09%)	2 (18.18%)	0 (0.00%)	0 (0.00%)	0 (0.00%)	0.306
Other	0 (0.00%)	0 (0.00%)	0 (0.00%)	1 (8.33%)	0 (0.00%)	0 (0.00%)	0.52

* Statistically significant; Chi-squared test or Fisher’s exact test.

**Table 10:** Age and cause of fracture.

Cause of Fracture	Age		*p*
	65–74 Years (*N* = 73)	75 Years and Older (*N* = 28)	
Falls	33 (45.21%)	15 (53.57%)	*p* = 0.826
Assault	9 (12.33%)	2 (7.14%)	
Traffic Accidents	7 (9.59%)	4 (14.29%)	
Work-related Injury	10 (13.70%)	2 (7.14%)	
Pathological /Iatrogenic Fractures	8 (10.96%)	3 (10.71%)	
Other	6 (8.22%)	1 (3.57%)	
No Data	0 (0.00%)	1 (3.57%)	

**Table 11:** Place of residence and cause of fracture.

Cause of Fracture	Place of Residence			*p*
	Small Town & Rural Areas (*N* = 51)	Medium-Sized Town (*N* = 14)	City (*N* = 36)	
Falls with Loss of Consciousness	5 (9.80%)	5 (35.71%)	5 (13.89%)	*p* = 0.009
Falls without Loss of Consciousness	11 (21.57%)	4 (28.57%)	18 (50.00%)	
Assault	6 (11.76%)	1 (7.14%)	4 (11.11%)	
Traffic Accident	6 (11.76%)	2 (14.29%)	3 (8.33%)	
Work-related Injury	12 (23.53%)	0 (0.00%)	0 (0.00%)	
Pathological/Iatrogenic Fracture	5 (9.80%)	2 (14.29%)	4 (11.11%)	
Other	6 (11.76%)	0 (0.00%)	1 (2.78%)	
No data	0 (0.00%)	0 (0.00%)	1 (2.78%)	

## Data Availability

The data that support the findings of this study are available from the corresponding author (P.M.), upon reasonable request.
